# 

**Published:** 1892-10-29

**Authors:** 


**Notices of Births, Deaths, and Marriages,** are inserted in this column at a charge of 2s. 6d. for an announcement not exceeding four lines.

### NOTICE TO CORRESPONDENTS.

All MS., Letters, Books for Review, and other matters intended for the Editor should be addressed The Editor, The Lodge, Porchester Square, London, W.

The Editor cannot undertake to return rejected M.S., even when accompanied by stamped directed envelope.

**Notice to Advertisers and Subscribers.**

All Advertisements, Orders for Copies of The Hospital, and Business Communications should be addressed to The Publisher, *not to the Editor*, The Hospital, 140, Strand, W.C.

The Cost of Subscription, post free, is as follows:—Per week, 2½d.; per quarter, 2s. 6d.; per half-year, 5s.; per annum, 8s. 6d. Foreign:—Per annum, 10s. 8d.; payable, in all cases, in advance.

**"Burdett's Hospital Annual," 1891—1892.**

Third year of publication. 600 pp. Boards, gilt lettering. A most complete guide to British and Colonial Hospitals, Dispensaries, Nursing and Convalescent Institutions, and Asylums. Price 3s. 6d. Published by The Scientific Press (Limited), 140, Strand, W.C.

**NOTICE.—The Index for Vol. XI. (ending March, 1892) is on sale at the Office of this Journal, price 2½d., post free.**

### BOOKS RECEIVED.

Simpkin, Marshall, and Co.

"Golden Rules of Surgical Practice." By E. H. Fenwick, F.R.C.S.I.

Ed. S. Livingstone.

"A Lecture Course of Elementary Chemistry." By H. T. Lilley, M.A.

"Catechism Series"—Chemistry, Part I. Inorganic.

Bailliere, Tyndall, and Cox.

"Keep Your Mouth Shut." By Fred. A. Smith, M.D.

### SCRAPS AND GLEANINGS.

The Hospital Saturday Fund for the present year now amounts to about £15,000.

The late Sir T. R. Edridge bequeathed the sum of £200 to the Croydon General Hospital.

Dr. Conway Evans, consulting physician at King's College Hospital, and medical officer to the Strand Board of Works, died last week.

The sum of £337 10s. 10d. has been raised by subscriptions, donations, and bazaar proceeds towards the Cottage Hospital for Mull.

The sum of £44 13s. has been handed over to the Basingstoke Cottage Hospital, being the amount collected on Hospital Sunday in that town.

The Chancellor of the Exchequer has forwarded the sum of £500 to the Royal College of Music, being the grant annually made to the College by Parliament.

The German Empress has ordered 50,000 marks of the proceeds of the Schloss Frieheit Lottery to be presented to the city of Berlin, for the benefit of poor mothers.

The Duke of Westminster has forwarded £600 to the funds of the Chester Infirmary, being the total fees paid during the past year for the privilege of viewing Eaton Hall.

Various improvements and alterations have been made to the Northampton General Infirmary during the past year. The total number of in-patients treated was 1,860, and out-patients 8,673.

The Rose-Innes Cottage Hospital for Marnoch and Forglen is to be opened this month. The hospital has been built with the money left by Miss Rose-Innes, of Natherdale, for its foundation and endowment.

It is reported that a man employed by an electric light company at Springfield, Mass., when working at the top of a high pole, grasped two wires charged with electricity, and, receiving a shock of 2,000 volts, was killed instantly.

The Duke of Westminster opened the new wing of the Chester Infirmary, which has been erected at a cost £2,500 as a memorial to the late Colonel Humberstone, who was for twenty-five years chairman to the Board of Management.

The Empress Frederick of Germany has promised fifty guineas to the Morell Mackenzie Memorial Fund. The memorial is to take the form of the extension of the Throat Hospital, Golden Square, to be called the "Morell Mackenzie Wing."

The City of Dublin Hospital is to be enlarged and entirely remodelled, thanks to the munificent contribution of £6,000 by Lord Pembroke. It is estimated that in addition to Lord Pembroke's gift £11,000 will be required to carry out the work satisfactorily.

A donation of £100 has been forwarded by Mr. F. Ravenscroft, Manager of the Birkbeck Bank, to the Metropolitan and City Police Orphanage, as a mark of appreciation for the special services rendered by members of the force during the recent run upon the bank.

The Lady Mayoress presented on Saturday at the Mansion House the prizes and certificates won by students attached to No. 2 District of the St. John Ambulance Association. The Lord Mayor mentioned that this centre alone contained 8,500 certificate holders capable of giving first aid to injured persons.

The new Surgical Pavilion of the Aberdeen Royal Infirmary contains accommodation for 145 patients, the wards are on three floors, and comprise six general wards, two eye wards, two lock wards, one ward for women, and several isolation rooms. On the ground floor are two large operating theatres, the ophthalmic department, and general offices. Electric light is used throughout the building.

The new Small-pox and Cholera Hospital for Kilmarnock has just been completed. The hospital contains two wards—a male and female—for six patients each. The administrative portion is connected with the wards by a corridor 18 ft. wide, but, at the same time, detached from the hospital proper. The wards internally are lined with white-enamelled bricks. The entire cost is about £1,200.

The Chairman and other members of the Committee of the Cancer Hospital (Free), Fulham-road, are appealing for the sum of £4,000 to enable them to make important and necessary improvements in the buildings. The Committee are anxious to meet the cost without encroaching on the ordinary resources of the Hospital. Donations will be thankfully acknowledged by the Secretary (Mr. W. H. Hughes).

The watch made for John Milton with raised figures on the dial so that the blind poet could read the time of day with his fingers, is, according to a Chicago newspaper, to be seen in a shop in that city. It was made by Thullier in Geneva in 1670. The story told is that it was pawned in St. Louis about a year ago by a gentleman of Verona, who exhibited a certificate of genuineness signed by the Curator of the British Museum.

The council of the University College Hospital have secured a site on which it is proposed to build an entirely new hospital after the most modern plan. The new hospital will obtain accommodation for 400 patients, and a portion of it will probably be erected in the rear of the present building ; there will therefore be no interruption in the reception of patients or the clinical work of the school, for as soon as the building is completed patients will be transferred to it.

The new Hospital at Droitwich, built by the generosity of Mrs. Dyson Perrins, of Malvern, contains on the ground floor a women's ward with five beds and another with three, a women's day-room and the men's day-room adjoining with a movable partition between, so that the two rooms may be thrown into one. The men's rooms at the other end of the building correspond with the women's; the kitchens and matron's room are on the same floor. The rooms on the upper floor correspond with those below.

The Bishop of Oxford has opened a new hospital for infectious diseases at Henley-on-Thames, which has been erected through the munificence of the late Mr. W. H. Smith. The ground on which it stands was presented by W. Dalziel Mackenzie, Esq. The hospital consists of four blocks ; first, the administrative block ; second contains four isolation wards, and is divided into two, so that it can be used for two distinct kinds of disease; third is for paying patients; fourth comprises ambulance, disinfecting houses, and mortuary.

### The Student of Medicine.

[All communications for this Supplement should be addressed to The Editor of The Hospital, at 140, Strand, with the words, "Students' Column," written in the left hand top corner of the envelope.]

### VACANCIES.

The following vacancies are announced this week, the amount of salary in each case being given after the name of the institution:

### Resident Medical Officers.

Birmingham General Dispensary, Resident Surgeon, doubly qualified—Salary £150 per annum, and £30 per annum allowance for cab hire. Applications to the Secretary by November 16th.

Bristol Eye Hospital, House Surgeon—Salary £120 per annum without board or residence. Applications to F. Richardson Cross by October 30th.

Crichton Royal Institution, Dumfries, Assistant Medical Officer; age from 25 to 28—Salary £200 per annum, with board and residence. Applications to Dr. Rutherford, Medical Superintendent.

Church of Scotland Jewish Mission, Medical Missionary for Mission and Hospital at Smyrna—Salary £300 per annum, with dwelling house. Applications to Rev. Dr. Alison, 1, South Lauder Road, or John A. Trail, W.S., 17, Duke Street, Edinburgh.

Devon County Lunatic Asylum, Assistant Medical Officer; unmarried—Salary £100 per annum, rising to £120, with board, lodging, and washing. Applications to A. E. Ward, Clerk to the Visitors, 9, Bedford Circus, Exeter, by October 28th.

Dover Hospital, House Surgeon—Salary £100 per annum, with furnished apartments, board, coals, light, and attendance. Applications to the Secretary, Mr. Edward Elwin, 13, Castle Street, Dover.

East London Hospital for Children, Glamis Road, Shadwell, E., House Physician; board and lodging provided. Applications to the Secretary by November 16th.

General Hospital, Barbadoes, West Indies, Junior House Surgeon—Salary £200 per annum, and quarters. Appointment for three years. Applications to the Secretary by December 1st.

London Throat Hospital, 204, Great Portland Street, W., House Surgeon. Appointment for six months. Honorarium at the rate of £50 per annum. Applications to the Secretary by October 27th.

Manchester Clinical Hospital for Women and Children, Park Place, Cheetham Hill Road, Manchester, House Surgeon—Salary £80 per annum, with apartments and board. Applications to Mr. Hubert Teague, 38, Barton Arcade, Manchester, by October 29th.

North West London Hospital, Kentish Town Road, N.W., Resident Medical Officer; appointment for six months—Salary £50. Applications to Alfred Craske, Secretary by October 28th.

Poplar Hospital for Accidents, Blackwall, E., Junior House Surgeon—Salary £50 per annum, with board, etc. Applications to the Secretary by October 27th.

Rotherham Hospital and Dispensary.—Assistant House Surgeon—Rooms, commons, and washing provided. Appointment for six months. Applications to the House Governor by October 26th.

Royal Free Hospital, Gray's Inn Road, W.C., Junior Resident Medical Officer, doubly qualified—Board and residence provided. Applications to the Secretary, C. W. Thiers, by October 24th.

Royal National Hospital for Consumption, Ventnor, Isle of Wight, Assistant Resident Medical Officer; unmarried—Salary £70 per annum. Applications to the Board of Management at the London Office, 34, Craven Street, Charing Cross, by November 3rd.

Smedley Hydopathic Establishment, Matlock, Derbyshire, Resident Physician; appointment for six months—Honorarium of 20 guineas. Board and residence free. Applications to the Secretary by November 5th.

Walsall Cottage Hospital, Resident Surgeon, doubly qualified—Salary £100 per annum, with board, lodging, and washing. Applications to the Chairman by October 22nd.

Weston-Super-Mare Hospital and Dispensary, Medical Officer to the Provident Dispensary attached to the Hospital; doubly qualified—Salary £60 per annum with board, lodging, and washing. Applications to the Honorary Secretary by October 26th.

### Non-Resident Medical Officers.

Birmingham General Hospital, Pathologist—Salary £120 per annum Applications to Howard J. Collins, House Governor, by November 4th.

Birmingham and Midland Free Hospital for Sick Children. Vacancy in the Medical Staff. Applications to the Medical Board, Children's Hospital, Steelhouse Lane, Birmingham, by November 8th.

Borough of Kingston-upon-Thames, Medical Officer of Health — Salary £150 per annum. Applications, endorsed "Medical Officer of Heath," to Harold A. Winser, Town Clerk, Clatten House, Kingston-on-Thames, by October 22nd.

Glamorgan County Council, County Medical Officer—Salary £750, of which £150 is intended to cover all travelling, office, and laboratory expenses. Applications to T. Mansel Franklin, Clerk, Glamorgan County Offices, Cardiff, by November 21st.

Hendon Local Board, Medical Officer of Health for the Urban Sanitary District and the Temporary Infectious Diseases Hospital—Salary as Medical Officer of Health, £125 per annum, and £35 (including the supply of medicines) for attending to patients at the hospital. Applications, endorsed "Medical Officer of Health," to Samuel Tilley, Clerk to the Board, Local Board Office, The Burroughs, Hendon, N.W., by October 29th.

Huddersfield Infirmary, Honorary Physician—Applications to Mr. F. Eastwood, Honorary Secretary, by November 1st.

THE STUDENT OF MEDICINE—(*continued*).

Infirmary for Consumption and Diseases of the Chest and Throat, 26, Margaret Street, Regent Street, W., two Visiting Physicians—Applications to W. H. Johnson, Secretary.

Leeds Friendly Societies' Medical Association, Assistant Medical Officer—Salary to commence, £120 per annum and midwifery fees. Applications to C. H. Wilson, 8, South Parade, Leeds, by October 25th.

Metropolitan Hospital, Kingsland Road, N.E., Surgeon; must be F.R.C.S.Eng. Applications to C. H. Byers, Secretary, by October 24th.

North Dublin Union, Finglas and Glasnevin Dispensary, Medical Officer—Salary. £170 per annum and fees. Applications to Mr. Thos. Conolly, Honorary Secretary, Fernville, Glasnevin. Election on October 31st.

North-West London Hospital, Kentish Town Road, N.W., Assistant Surgeon; must be F.R.C.S.Eng. Applications to A. Craske, Secretary, by October 31st.

Sheffield School of Medicine, Tutor, to take charge of dissecting-rooms and hold classes in anatomy and physiology—Salary, commencing at £100 per annum. Applications to the Secretary by October 25th.

